# Post COP26: legal action now part of public health’s environment and climate change toolbox

**DOI:** 10.1093/eurpub/ckac057

**Published:** 2022-05-31

**Authors:** David W Patterson, Richard Harvey, Vlatka Matkovic, Marlies Hesselman, Farhang Tahzib

**Affiliations:** Groningen Centre for Health Law, , Department of Transboundary Legal Studies, Faculty of Law, University of Groningen, Groningen, Netherlands; Legal Unit, , Greenpeace International, Amsterdam, Netherlands; Garden Court Chambers, London, England; Health and Energy, , Environment & Health Alliance (HEAL), Brussels, Belgium; Groningen Centre for Health Law, , Department of Transboundary Legal Studies, Faculty of Law, University of Groningen, Groningen, Netherlands; Faculty of Public Health, London, UK

Public health practitioners have long relied on good science to make the case for sound public policies. In the climate domain, the *Lancet Countdown* reports meticulously document the threats to public health of inaction on global warming.^1^ The WHO and the UN Intergovernmental Panel on Climate Change have long recognized the impacts on health as wide ranging, diverse and overwhelmingly negative.^2^

We are all aware of the physical and mental health impacts of climate change. There is a plethora of government promises, coupled with companies telling us they are developing technical solutions to address our warming planet—but governments are not responding with the needed urgency. Ballot box pressure (even in democracies) does not guarantee results. Industry greenwashing and shorter-term concerns distract from our overheating planet. Beyond describing the threats in ever greater detail, what more can the public health community do?

The environmental movement has long used legal action to challenge polluters and government inaction or collusion. The health community can lend its support. At the least, litigation pushes the issues further into the public domain and can force governments and companies to respond. Courts can also issue injunctions to stop ongoing harms, require governments to review permits afresh, order both compensation and, where possible, repairs to damaged environments at the polluters’ expense. Most important are the opportunities generated for public scrutiny and debate.

Legal systems are as varied as health systems, yet there are principles common to all jurisdictions. The choice of legal forum is key—it could be under a national constitutional protection of the right to life, a human rights treaty or environmental or tort law. In any court action, legal ‘standing’ and evidence are essential. Sound scientific evidence is as critical to successful litigation as to effective public health policies.

Increasingly, public health practitioners are asked to testify in court about the known health impacts of environmental harms. Collecting this evidence requires foresight, meticulous record-keeping, peer support, and the courage to withstand questioning of professional capacity. The causal link must be made, and expert testimony will be decisive. It took Sir Stephen Holgate 18 months to compile the evidence that air pollution was a causal factor in the death of 9-year-old Ella Adoo-Kissi-Debrah in London. In 2021, the UK government announced measures to tackle air pollution after a coroner ordered that Ella’s death certificate to be amended to note toxic air contributed to her death. The partnership between the legal team, the scientist and Ella’s mother, Rosamund Adoo-Kissi-Debrah was key in the coroner reaching this decision.^3^

In October 2021, EUPHA co-hosted a dialogue between public health and environmental advocates on the use of litigation to address our common goals.^4^ Dr Maria Neira, WHO’s Director, Environment, Climate Change and Health noted the crucial role for public health specialists as *experts* in litigation—building on the experience of tobacco and asbestos. She urged nurses and doctors to collect and record evidence in medical files which can later be used in expert testimony.

Environmental rights are human rights, as regional human rights systems are increasingly recognizing. Since 2020, four cases have been filed with the European Court of Human Rights in Strasbourg on States’ responsibilities for the physical or psychological impacts of climate change on human health. In Switzerland, senior women claimed they are particularly vulnerable to climate change impacts and that their government’s policies were inadequate to meet Paris Agreement obligations.^5^ In Norway, youth climate activists have challenged their government’s decision to grant oil and gas exploration licences in the fragile Arctic.^6^ In Portugal, six young people have accused 33 European countries of failing to do their part to avert climate catastrophe.^7^ Finally, in Austria, a man with temperature-sensitive multiple sclerosis has asked the Court for urgent protection against the severely debilitating effects of each incremental degree of warming and longer heat waves.^8^

With these four cases, the European Court will be asked to issue landmark decisions on climate justice. The Inter-American Court of Human Rights has already recognized the right to a healthy environment, including the ‘rights of nature’. The United Nations Human Rights Council similarly endorsed the right to a healthy environment in 2021, and established a Special Rapporteur for climate and human rights.^9^ Judgments from supra-national courts may well inspire domestic judges to fashion appropriate remedies for climate injustice.

Even a partial victory can set a precedent for the next legal challenge. Litigating strategically can bring wins for individual applicants, but also helps set out the law, or highlight gaps in legal protection. In October 2021, the UN Committee on the Rights of the Child rejected a climate complaint by 16 children against Argentina, Brazil, France, Germany and Turkey on procedural grounds. Yet, the Committee decided that in principle a State party can be held responsible for the negative impact of its carbon emissions on the rights of children, both within and outside its territory.^10^ The decision opens the door to further challenges by children to government inaction.

The Preamble and Article 13 of the Paris Agreement affirm the importance of public awareness, public participation, and public access to information. However, greater civil society engagement is needed. By contrast, in 2001, at the UN General Assembly’s first-ever Special Session on a public health issue, States were urged to include people living with HIV in their national delegations, and many did so.

In Europe, the legal framework for diverse participation in the development of environmental and climate policies already exists. In 2014, the Economic Commission for Europe issued comprehensive recommendations under the Aarhus Convention on promoting effective public participation in decision-making in environmental matters. Let us now measure the inclusion of public health experts and representatives of indigenous communities and children in government national roundtables and official delegations to UN climate events—such as at COP27—the Conferences of Parties meeting in Sharm el Sheik, Egypt in November 2022. Bridges between the public health, environmental and legal communities and advocates must be strengthened if we are to ‘keep 1.5 alive’.

*Conflicts of interest*: The authors declare no conflict of interest.
